# Investigation of Antioxidant Interactions between Radix *Astragali* and *Cimicifuga foetida* and Identification of Synergistic Antioxidant Compounds

**DOI:** 10.1371/journal.pone.0087221

**Published:** 2014-01-30

**Authors:** Fei Wang, Shancang Zhao, Feng Li, Bo Zhang, Yi Qu, Tianlei Sun, Ting Luo, Dapeng Li

**Affiliations:** 1 Department of Food Science, Shandong Agricultural University, Taian, Shandong, China; 2 Shandong Provincial Key Laboratory of Test Technology on Food Quality and Safety, and Central Laboratory, Shandong Academy of Agricultural Sciences, Jinan, Shandong, China; University of Sassari, Italy

## Abstract

The medicinal plants of Huang-qi (Radix *Astragali*) and Sheng-ma (*Cimicifuga foetida*) demonstrate significantly better antioxidant effects when used in combination than when used alone. However, the bioactive components and interactional mechanism underlying this synergistic action are still not well understood. In the present study, 2,2-diphenyl-1-picrylhydrazyl (DPPH) radical scavenging assay was employed to investigate the antioxidant capacity of single herbs and their combination with the purpose of screening synergistic antioxidant compounds from them. Chromatographic isolation was performed on silica gel, Sephadex LH-20 columns and HPLC, and consequently to yield formononetin, calycosin, ferulic acid and isoferulic acid, which were identified by their retention time, UV λ_max_, MS and MS/MS data. The combination of isoferulic acid and calycosin at a dose ratio of 1∶1 resulted in significant synergy in scavenging DPPH radicals and ferric reducing antioxidant power (FRAP) assay. Furthermore, the protective effects of these four potential synergistic compounds were examined using H_2_O_2_-induced HepG2 Cells bioassay. Results revealed that the similar synergy was observed in the combination of isoferulic acid and calycosin. These findings might provide some theoretical basis for the purported synergistic efficiency of Huang-qi and Sheng-ma as functional foods, dietary supplements and medicinal drugs.

## Introduction

Traditional herbs in Asia are widely applied to medical treatments and as dietary supplements. Many of these edible and medicinal plants are excellent source of phytochemicals, such as phenolic acids and flavonoids, which have more potent antioxidant activity than common dietary [1], [2]. However, accumulated evidence show that an individual isolated ingredient commonly exhibited poorer antioxidant activity than a whole or partially purified extract of a plant [3], [4], suggesting that the isolation and purification of phytochemicals might lead to the reduction of their antioxidant ability. Thus, synergistic interactions between phytochemicals are of vital importance for the whole antioxidant efficacy of a plant.

Free radical scavenging effect is one kind of the most important antioxidant abilities of phytochemicals. Various free radicals that occurred in body can cause damage to cells, injure the immune system and lead to a series of chronic degenerative diseases such as heart disease, Alzheimer’s disease and cancer [5]. Thus, the study on synergistic interactions between phytochemicals which possess free radical scavenging ability tends to get more and more attention. Alpha-tocopherol was found to show potent synergistic action in scavenging radicals when used in combination with proanthocyanidins [6] and quercetin [7]. The study on the scavenging capacity and synergistic effects of lycopene, vitamin E, vitamin C, and β-carotene against the 2,2-diphenyl-1-picrylhydrazyl (DPPH) free radicals revealed that the combination of lycopene, vitamin E and vitamin C resulted in significant synergism in the three-component system [8]. Hugo et al. found a synergistic effect in the majority of the all combinations of phenolics (chlorogenic, gallic acid, protocatechuic and vanillic acid) using the DPPH assay [9]. However, most of these present researches commonly confined to some specific compounds, especially the vitamin C and vitamin E, the synergistic interactions between phenolics were rarely reported.

Traditional herbs are commonly used in the form of combination. Herb pair is a basic unit of multi-herb recipes [10], which consists of two single herbs and presents significantly better pharmacological efficacy than individual herbs [11]. Practitioners always believed that synergistic interactions between the components of herbals greatly contribute to the enhancement of their therapeutic efficacy. And they also have made great efforts in screening of active compounds from various herbals. However, the target object of these researches was generally one kind of plant, few studies kept a watchful eye on the screening and identification of synergistic components from two or more herbals. Huang-qi (Radix *Astragali* ) is the root of *Astragalus membranaceus* (Fisch.) Bunge and/or *A. membranaceus* var. *mongholicus* (Bunge) Hsiao. It is commonly used as food supplement on the western market and as diuretics in East Asia [12]. Sheng-ma (rhizome of *Cimicifuga foetida* L.) has been widely applied as a cooling and detoxifying agent and for alleviation of fever, pain, and inflammation [13]. The herb pair of Huang-qi and Sheng-ma is one of the most widely used combinations in traditional Chinese medicine. Our previous study (unpublished data) has found that the ethanol extract of Huang-qi and Sheng-ma exhibited synergistic antioxidant activity when used in combination. In the present study, the synergistic antioxidant compounds from these two edible and medicinal plants were investigated, which may provide some theoretical basis for the purported synergistic efficiency of Huang-qi and Sheng-ma as functional foods, dietary supplements and medicinal drugs.

## Materials and Methods

### Materials

The herb plant materials of Huang-qi (*Astragalus membranaceus* var. *mongholicus* (Bunge) Hsiao) (AME) and Sheng-ma (rhizome of *Cimicifuga foetida* L.) (CFO) were purchased from the Shijiazhuang Pharmaceutical Group of China and authenticated by Professor Lingchuan Xu, Shandong University of Traditional Chinese Medicine, P. R. China.

### Chemicals

Folin-Ciocalteu’s phenol reagent, 2,2-diphenyl-1-picrylhydrazyl (DPPH), 2,4,6-tripyridyl-s-triazine (TPTZ) and 2,2-azinobis (3-ethyl-benzothiazoline-6-sulfonicacid) (ABTS) were purchased from Sigma (St. Louis, MO); Calycosin (purity, ≥98%), formononetin (purity, ≥98%) were purchased from Mairier company (Shanghai, China); Ferulic acid (purity, ≥98%) and isoferulic acid (purity, ≥98%) were purchased from Yuanye Company (Shanghai, China); Fetal bovine serum (FBS), Dulbecco’s modified Eagle’s minimum essential medium (DMEM) and Trypsin-EDTA (0.25% trypsin with EDTA-4Na) were purchased from Gibco (Grand Island, NY); The mixture of penicillin and streptomycin, 3-(4,5-dimethylthiazol-2-yl)-2,5-diphenyl-tetrazolium bromide (MTT) were obtained from Solarbio (Beijing, China); HPLC-grade acetonitrile was obtained from Fisher Scientific (Waltham, MA); Water was purified using a Milli-Q system from Millipore (Bedford, MA).

### Determination of Antioxidant Activity

#### DPPH free radical scavenging assay

The DPPH radical scavenging assay used in this study was slightly modified on the basis of previous reports [14], [15]. Briefly, 0.1 mL of samples of different concentrations was added to 3.9 mL of DPPH solution in ethanol (0.1 mmol/L) and mixed immediately. After reacting at 37°C for 60 min, the absorbance was measured at 517 nm, and the antioxidant capability (AA) was expressed as the percentage of DPPH? reduced, which was calculated with the following formula:
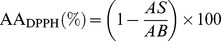



Where A_S_ was the absorbance of the DPPH solution after reacting with the sample at a given concentration and A_B_ was the absorbance of the DPPH• solution with an ethanol blank instead of the sample. The percentage of DPPH? reduced was plotted against the concentration of each sample, and an IC_50_ value, which is defined as the concentration of the sample needed to scavenge 50% of the DPPH?, was calculated from the graph.

#### ABTS free radical scavenging assay

The ABTS free radical scavenging assay was based on previous method with a few of modifications [16]. Potassium persulfate was added into 7 mmol/L of ABTS?^+^ and kept for 12–16 h at room temperature in the dark environment. Then the ABTS?^+^ solution was diluted with ethanol to an absorbance of 0.70±0.02 at 734 nm before analysis. 0.1 mL of samples of different concentrations was added to 3.9 mL of ABTS?^+^ solution in ethanol and mixed immediately. The absorbance of the mixture was measured at 734 nm after reaction for 15 min at room temperature, and the antioxidant capability (AA) was expressed as the percentage of the reduced ABTS?^+^. The calculation method is similar to DPPH free radical scavenging assay.

#### Ferric Reducing Antioxidant Power (FRAP) assay

The FRAP working solution was prepared freshly as previously described with slight modifications [17]. Briefly, it was the mixture of acetate buffer (300 mmol/L, pH 3.6), TPTZ solution (10 mmol/L in 40 mmol/L HCl) and FeCl_3_?6H_2_O solution (20 mmol/L) in a proportions of 10∶1:1. 0.1 mL of samples in ethanol was added directly to 3.9 mL of FRAP working solution. The absorbance of the mixture was measured at 593 nm after 10 min of reaction. The calibration curve was constructed with aqueous solutions of FeSO_4_?7H_2_O (100–1000 µmol/L), and the results were expressed as mmol Fe(II)/g dry weight of herb extract.

### Determination of Total Phenolics and Flavonoids Content

Total phenolics content was determined using the Folin-Ciocalteu method with some modifications [18]. Briefly, 0.1 mL of sample was mixed with 1 mL of the Folin-Ciocalteu working solution (diluted ten-fold) and incubated at room temperature for 5 min, then 1 mL of Na_2_CO_3_ solution(0.1 g/mL) was added to the mixture. After incubation for 90 min at room temperature, the absorbance of sample was measured at 765 nm, and the results were expressed as gallic acid equivalents per gram sample (mg GAE/g).

Total flavonoids content of the samples was measured according to previous colorimetric assay with some modifications [19]. 0.1 mL sample was mixed with 0.3 mL of NaNO_2_ solution (0.05 g/mL) and incubated for 5 min, then 0.3 mL of AlCl_3_ solution (0.1 g/mL) was added and incubated for another 6 min. The reaction was terminated by adding 2 mL of NaOH solution (1 mol/L). Then absorbance of the mixture was measured at 510 nm immediately. The results were expressed as rutin equivalents per gram sample (mg RE/g).

### Determination of Protective Effect against H_2_O_2_-induced Oxidative Damage in HepG2 Cells

#### Cell culture

HepG2 cell was obtained from American Type Culture Collection (ATCC, Rockville, MD) and incubated in DMEM supplemented with heat-inactivated 10% FBS, 100 U/mL penicillin, 100 µg/mL of streptomycin in a humidified atmosphere of 5% CO_2_ at 37°C, and the medium was changed every other day.

#### Cell viability assay

Cells were seeded in 96-well plates at a density of 1×10^4^ cells/well and incubated for 24 h. Thereafter, the medium was replaced with fresh medium containing various concentrations of tested compounds for another 24 h–incubation prior to exposure to 1.4 mmol/L H_2_O_2_ for 4 h. At the end of the incubation period, the medium was decanted, and 100 µL of MTT dye solution (0.5 mg/mL in PBS) were added into each well, and the plates were incubated for 4 h at 37°C. Then, 150 µL of DMSO was added to dissolve/extract tetrazolium dye. Relative cell viability was calculated by determining the absorbance at 570 nm and untreated control cells were assigned a relative viability of 100%.

### Evaluation of Synergistic Effect

In chemical-based assays, the herb pair was consisted of two single herbs at a dose ratio of 1∶1. Its scavenging ability against free radicals was defined as Herb pair IC_50_. In cell-based assays, the two drugs in the combination were not at a constant ratio. The synergistic effect of the herb pair was evaluated using CalcuSyn software, which was developed for dose-effect analysis in drug-combination studies. The combination index (CI) was expressed as CI_25_, CI_50_ and CI_75_ when the fraction affected (Fa) by the dose was 0.25, 0.50, 0.75, respectively. CI ≤0.90, 0.90< CI <1.10 or CI ≥1.10 represent synergistic, additive, and antagonistic effects, respectively.

In the FRAP assay, the theoretical value was calculated as the sum of two single herbs’ values multiplying 0.5 for their proportion in herb pair was 0.5∶0.5. When the actual value of herb pair was significantly larger than their theoretical value (*P*<0.05), the synergistic effect was sure to exist in the combination.

### Preparation of Crude Extracts

The powder of single herbs (AME, CFO, 500 g) was extracted twice with 2.5 L of 95% of ethanol by soxhlet extractor and each time lasted 2.5 h. The extracts then were merged, concentrated by rotary evaporator to obtain the residues. The residues were re-suspended in appropriate volume of deionized water and successively extracted with equivalent volume of petroleum ether (PE), chloroform (CF), ethyl acetate (EA), and n-butyl alcohol (NB), giving four fractions for each herb extract (AME-PE, AME-CF, AME-EA, AME-NB) and (CFO-PE, CFO-CF, CFO-EA, CFO-NB). The crude extracts were stored at −20°C until use.

### Separation and Purification of Crude Extracts

The samples of AME-CF (9.0 g) and CFO-CF (3.5 g) were respectively subjected to silica gel column chromatography to isolate the active ingredients. AME-CF was eluted in the following order by petroleum ether-acetone (16∶4, 15∶5, 14∶6, 11∶9, v/v), chloroform-methanol (18∶2, 17∶3, 16∶4, 15∶5, 13∶7, 0∶20, v/v). Ten fractions were collected according to thin-layer chromatography (TLC). After being stored at 4°C for some time, precipitation appeared in fraction 2 (0.407 g) and fraction 4 (0.258 g), then needle-like white crystallization (3.5 mg) was obtained by re-crystallization from fraction 4, while the yellow precipitation of fraction 2 did not change after re-crystallization. The fraction 3 (0.208 g) and fraction 5 (0.209 g) were purified by Sephadex LH-20 chromatography using chloroform-methanol solution as the eluent. The eluent for separating CFO-CF was chloroform-methanol (19∶1-18∶2-17∶3-16∶4-10∶10-0∶20, v/v). After TLC analysis, four fractions were obtained. Fraction 1 (0.793 g) was repeatedly subjected to Sephadex LH-20 chromatography for purification.

### Preparation of Standard Solutions

3.1 mg of ferulic acid and 4.1 mg of isoferulic acid were weighed, and dissolved in 1 mL of methanol respectively to yield the stock solutions. 200 µL of ferulic acid stock solution and 200 µL of isoferulic acid stock solution were mixed with 600 µL of methanol to obtain the mixed standard solution (ferulic acid 0.62 mg/mL, isoferulic acid 0.82 mg/mL). 5.0 mg of calycosin and 2.4 mg of formononetin were weighed, and dissolved in 1.5 mL of methanol respectively. 60 µL of calycosin solution and 60 µL of formononetin solution were mixed with 480 µL of methanol to obtain the mixed standard solution (calycosin 0.33 mg/mL, formononetin 0.16 mg/mL).

### HPLC Conditions

The Waters Acquity Ultra Performance LC system (Waters Corporation, Milford, MA, USA) consisting of binary solvent manager, an auto-sampler, column manager, and a diode-array detector (DAD) was used for setting the reverse-phase liquid chromatographic conditions (data analysis software Empower). Chromatographic separations were carried out using an Atlantis ®dC18 column (250 mm×4.6 mm, 5 µm, Waters). HPLC separation was performed using the linear gradient at 30°C and the flow rate of 0.8 mL/min. The injection volume was 10 µL. Detection wavelength was set at 254 nm and UV spectra from 200 to 500 nm were also recorded for peak identification.

For the samples of AME, the mobile phase consisted of water (A) and acetonitrile (B) using the elution gradient. The gradient program was adopted as follows: Isocratic elution of 10% B over the first 5 min, linear from 10 to 20% B (5–10 min), linear from 20 to 35% B (10–20 min), linear from 35 to 45% B (20–30 min), linear from 45 to 55% B (30–40 min), linear from 55 to 65% B (40–50 min), linear from 65 to 10% B (50–55 min), held for 5 min until the separation finished. For the samples of CFO, the mobile phase was 0.1% of phosphoric acid in water (A) and acetonitrile (B) using the elution gradient. The gradient program was adopted as follows: Isocratic elution of 20% B over the first 10 min, linear from 20 to 30% B (10–20 min), linear from 30 to 35% B (20–30 min), linear from 35 to 40% B (30–40 min), linear from 40 to 20% B (40–45 min), held for 5 min until the separation finished.

### Mass Spectrometry

The Waters Micromass Quattro Ultima PT system (Waters Corporation, Milford, MA, USA) equipped with the electrospray ionization (ESI) source was used for mass spectrometric measurement (data analysis software MassLynx 4.0 SP4). The column, mobile phase and elution program were transferred directly from the system described in HPLC method to this system excepted for the mobile phase for samples of CFO. Since phosphoric acid would seriously damage the mass spectrometric, the 0.1% of phosphoric acid in water was replaced by 0.1% of formic acid in water. The injection volume was 5 µL. In order to adapt to the requirement of ESI-MS/MS, a post-column flow splitter (split ratio 1∶1) was connected to the C18 column, and the flow rate of 0.8 mL/min was reduced to 0.4 mL/min before the elution buffer entered the ESI interface. The mass spectrometry detector (MSD) parameters were as follows: positive ion mode; capillary voltage, 3.5 KV; cone voltage, 45.0 V; collision energy, 10.0–25.0 eV; source temperature, 110°C; desolvation temperature, 400°C; cone gas (argon, 99.9999% purity) flow rate, 80 L**·**h^−1^; desolvation gas (nitrogen, 99.9% purity) flow rate, 562 L/h. Mass analyzer scanned from 100 to 1000 u. The MS/MS spectra were recorded in auto-MS/MS mode.

### Statistical Analysis

All experiments were carried out in triplicate and the data were expressed as mean ± standard deviation. Statistical analysis was performed with the one-way analysis of variance (ANOVA), the Duncan test and the Bivariate Correlations using the SPSS 16.0 software. Values of *P*<0.05 were considered to be statistically significant.

## Results

### Antioxidant Activity of AME, CFO and Different Solvent–extracted Fractions

**Figure 1:**
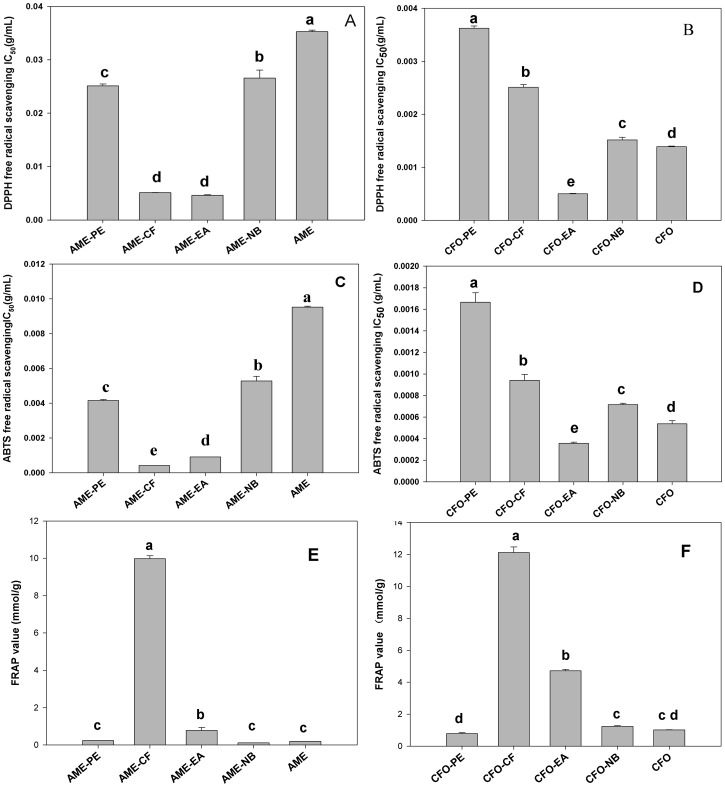
The antioxidant abilities of AME and CFO and different solvent-extracted fractions. (A): DPPH free radical scavenging ability of AME and four solvent-extracted fractions; (B): DPPH free radical scavenging ability of CFO and four solvent-extracted fractions; (C): ABTS free radical scavenging ability of AME and four solvent-extracted fractions; (D): ABTS free radical scavenging ability of CFO and four solvent-extracted fractions; (E): FRAP value of AME and four solvent-extracted fractions; (F): FRAP value of CFO and four solvent-extracted fractions.

As shown in Fig. 1, compared with other solvent-extracted fractions of AME, the chloroform extracts and ethyl acetate extracts showed higher scavenging efficacy against DPPH free radicals. In addition, AME-CF owned the strongest ABTS free radical scavenging ability and ferric reducing antioxidant power, which was basically in consistent with the previous study [20]. Among the different solvent-extracted fractions of CFO, the ethyl acetate extracts presented the strongest scavenging ability against DPPH and ABTS free radicals, while the chloroform extracts had the highest FRAP value. Thus, AME-CF, AME-EA, CFO-CF and CFO-EA were selected for further analysis.

### Total Phenolics and Flavonoids Content of AME, CFO and Different Solvent-extracted Fractions

Phenolic and flavonoid compounds are important antioxidants in many plants. Thus, their contents in AME, CFO and different solvent-extracted fractions were determined. From Table 1, it was found that the phenolics and flavonoids content of CFO was significantly higher than that of AME, which might be responsible for the stronger antioxidant activity of CFO. Among the solvent-extracted fractions of AME, AME-CF owned the highest phenolics and flavonoids content. In the case of CFO, the highest phenolics and flavonoids content was observed in CFO-EA.

**Table 1:** Total phenolic and flavonoid contents of AME, CFO and different solvent-extracted fractions.

	AME	AME-PE	AME-CF	AME-EA	AME-NB	CFO	CFO-PE	CFO-CF	CFO-EA	CFO-NB
**Phenolics** **content** **(mg GAE/g)**	22.193±0.463	17.046±0.420	91.782±2.628	76.784±4.409	16.806±0.267	108.85±3.519	69.702±4.235	143.98±3.892	319.82±1.638	97.047±3.464
**Flavonoids** **content** **(mg RE/g)**	35.550±0.556	76.169±0.519	95.653±2.006	52.513±0.796	14.584±0.200	187.30±3.582	130.92±2.778	160.36±7.572	384.79±6.976	91.670±2.398

### The Correlation between the Phenolic/Flavonoid Increments and Antioxidant Activity

**Figure 2:**
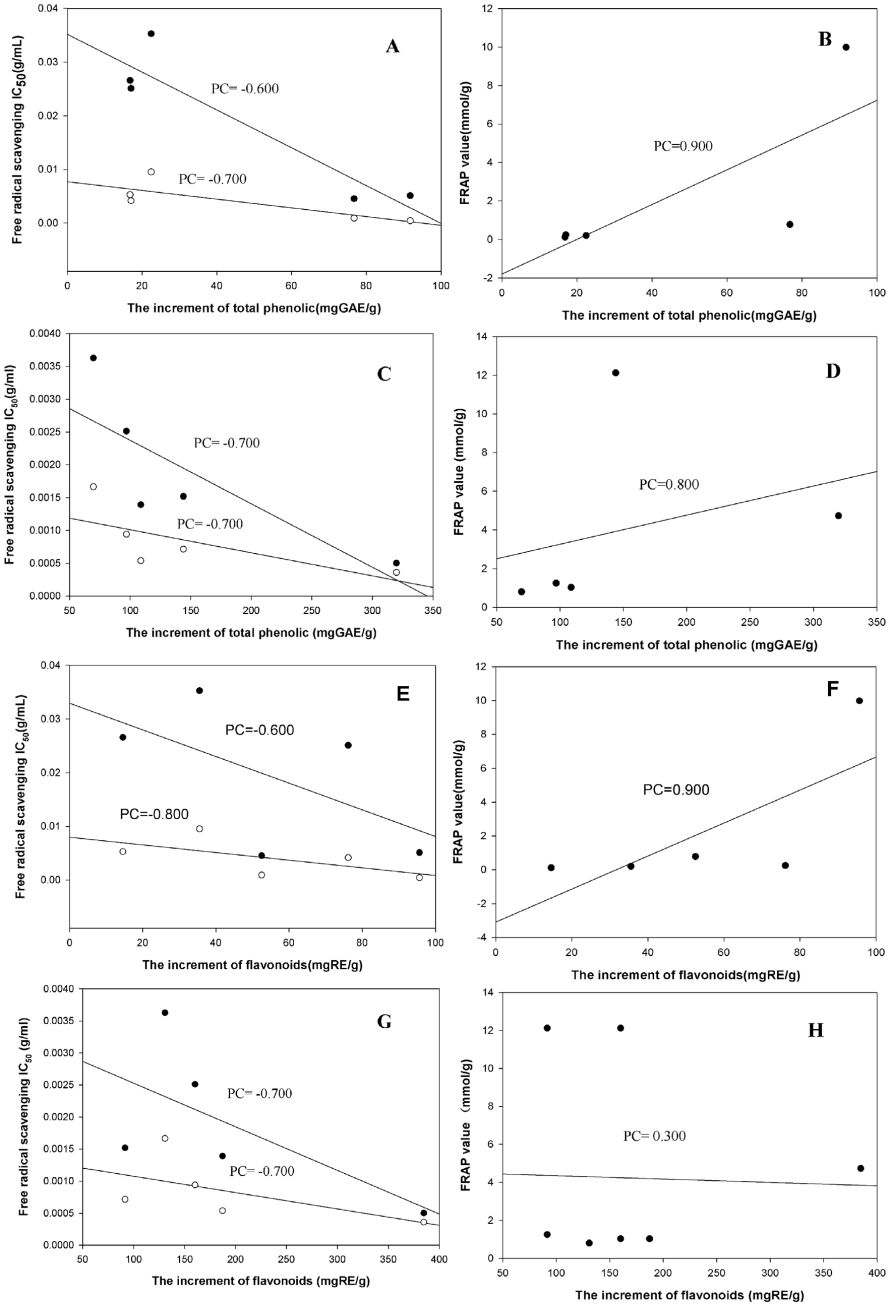
The correlation between the increments of phenolics/flavonoids and antioxidant activities. (A): •The correlation between the increment of total phenolics and DPPH scavenging IC_50_ in AME; ○The correlation between the increment of total phenolics and ABTS scavenging IC_50_ in AME. (B): The correlation between the increment of total phenolics and FRAP value in AME. (C): •The correlation between the increment of total phenolics and DPPH scavenging IC_50_ in CFO; ○The correlation between the increment of total phenolics and ABTS scavenging IC_50_ in CFO. (D): The correlation between the increment of total phenolics and FRAP value in CFO. (E): •The correlation between increment of flavonoids and DPPH scavenging IC_50_ in AME; ○The correlation between the increment of flavonoids and ABTS scavenging IC_50_ in AME. (F): The correlation between the increment of flavonoids and FRAP value in AME. (G): •The correlation between the increment of flavonoids and DPPH scavenging IC_50_ in CFO; ○The correlation between the increment of flavonoids and ABTS scavenging IC_50_ in CFO. (H): The correlation between the increment of flavonoids and FRAP value in CFO. PC means Spearman Correlation Coefficient.

Phenolic substances play a very important role in antioxidant effects [21]. Fig. 2 showed the Spearman correlation coefficients (PC) between the total phenolic/flavonoid increments and antioxidant activity increments determined via three anti-oxidative assays. AME-PC_DPPH_: 0.6/0.6, AME-PC_ABTS_: 0.7/0.8, CFO-PC_DPPH_: 0.7/0.7 and CFO-PC_ABTS_: 0.7/0.7 were ≥0.5, suggesting that the phenolic/flavonoid increments of different extracts were closely related to the enhancement of free radical scavenging abilities. Besides, there was significant linear correlation between the phenolic/flavonoid increments and FRAP values increments as indicated by the correlation coefficient of 0.9* in various extracts of AME, suggesting that the phenolic/flavonoid substances in AME played an important role in the ferric reducing antioxidant activity. However, CFO-PC_FRAP_ was 0.8/0.3, mean that the active substances that greatly contributed to the ferric reducing antioxidant capacity of CFO were phenolics, not flavonoid compounds.

### Screening of Synergistic Antioxidant Fractions from Active Solvent-extracted Fractions

DPPH free radical scavenging efficacy of the combinations (AME-CF+CFO-CF, AME-CF+CFO-EA, AME-EA+CFO-CF, AME-EA+CFO-EA) was measured to examine their synergistic effects. As shown in Table 2, only (AME-CF+CFO-CF) and (AME-EA+CFO-CF) pairs showed synergistic action at the low, moderate and high dose. Since the synergistic effect of (AME-CF+CFO-CF) pair (CI_50_, 0.49) was stronger than that of (AME-EA+CFO-CF) pair (CI_50_, 0.53), the active solvent-extracted fractions of AME-CF and CFO-CF were selected for further isolation and analysis.

**Table 2:** DPPH radical scavenging ability and synergistic effect for combinations of different solvent-extracted fractions.

Samples	Herb pair IC_50_(mg/mL)	CI_25_	CI_50_	CI_75_
AME-CF+CFO-CF	1.706	0.543	0.494	0.449
AME-CF+CFO-EA	1.727	1.956	1.917	1.881
AME-EA+CFO-CF	1.715	0.581	0.526	0.477
AME-EA+CFO-EA	1.526	0.355	2.144	12.986

### Screening of Active Fractions from AME-CF and CFO-CF

**Figure 3:**
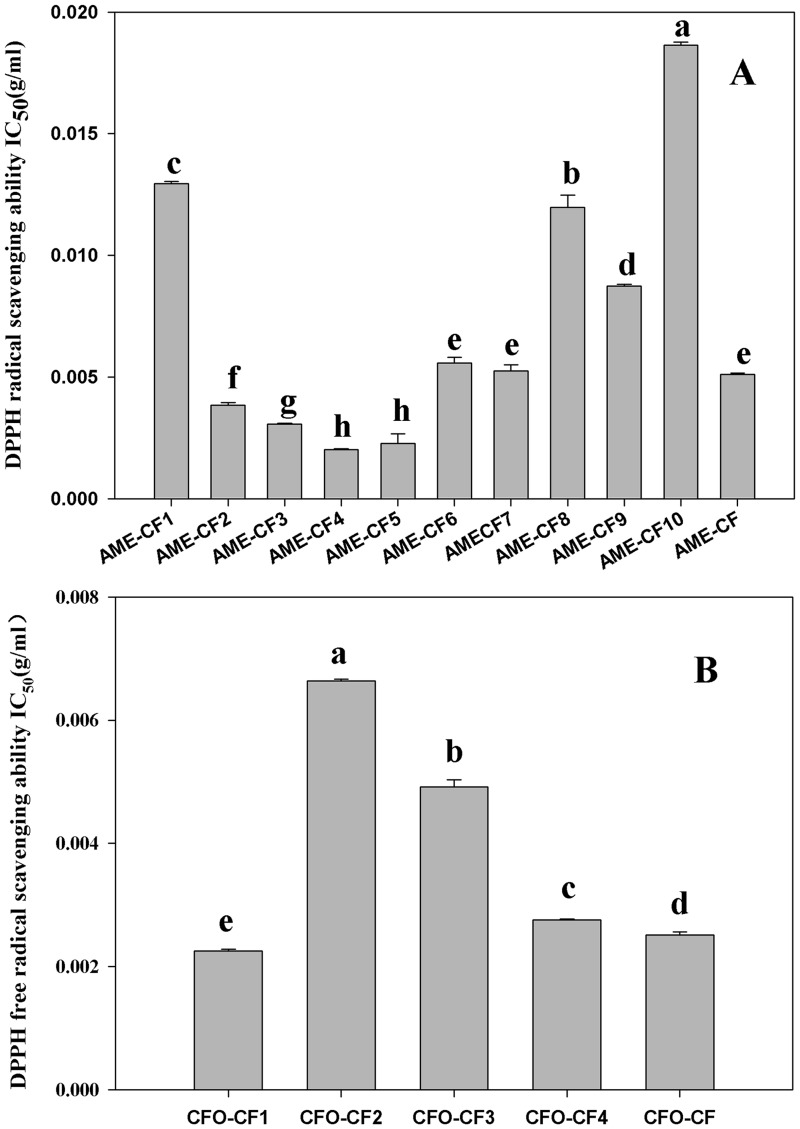
The DPPH radical scavenging ability of different fractions isolated from AME-CF and CFO-CF. A: The DPPH radical scavenging ability of 10 fractions isolated from AME-CF. B: The DPPH radical scavenging ability of 4 fractions isolated from CFO-CF.

Ten fractions isolated from AME-CF and four fractions isolated from CFO-CF were examined for their scavenging abilities against DPPH free radicals, and the results were shown in Fig. 3. Among the ten fractions isolated from AME-CF, fraction 2, 3, 4 and 5 showed higher free radical scavenging efficacy than other fractions. Moreover, these fractions exhibited higher potency in scavenging DPPH radicals than the original AME-CF sample (*P*<0.05). Among the four fractions isolated from CFO-CF, fraction 1 showed the strongest free radical scavenging ability compared with other fractions and the original CFO-CF sample. Thus, AME-CF_2_, AME-CF_3_, AME-CF_4_, AME-CF_5_ and CFO-CF_1_ were selected for the further examination of synergistic effects after purified.

### Screening and Identification of Potential Synergistic Antioxidant Compounds

AME-CF_2_, AME-CF_3_, AME-CF_4_ and AME-CF_5_ were paired with CFO-CF_1_ to examine the possible synergistic antioxidant efficacy, respectively. As shown in Table 3, (AME-CF_2_+CFO-CF_1_) pair and (AME-CF_4_+CFO-CF_1_) pair resulted in synergistic effects. However, comparing with (AME-CF+CFO-CF) pair (CI_50_, 0.49), the synergy weakened to some extent along with the isolation and purification.

**Table 3:** The DPPH radical scavenging ability and synergistic effect for combinations of different fractions isolated from AME-CF and CFO-CF.

Samples	Herb pair IC_50_(mg/mL)	CI_25_	CI_50_	CI_75_
AME-CF_2_+CFO-CF_1_	2.860	0.824	0.848	0.879
AME-CF_3_+CFO-CF_1_	3.404	0.882	0.918	0.962
AME-CF_4_+CFO-CF_1_	1.501	0.397	0.545	0.756
AME-CF_5_+CFO-CF_1_	2.861	1.109	1.100	1.105

**Figure 4:**
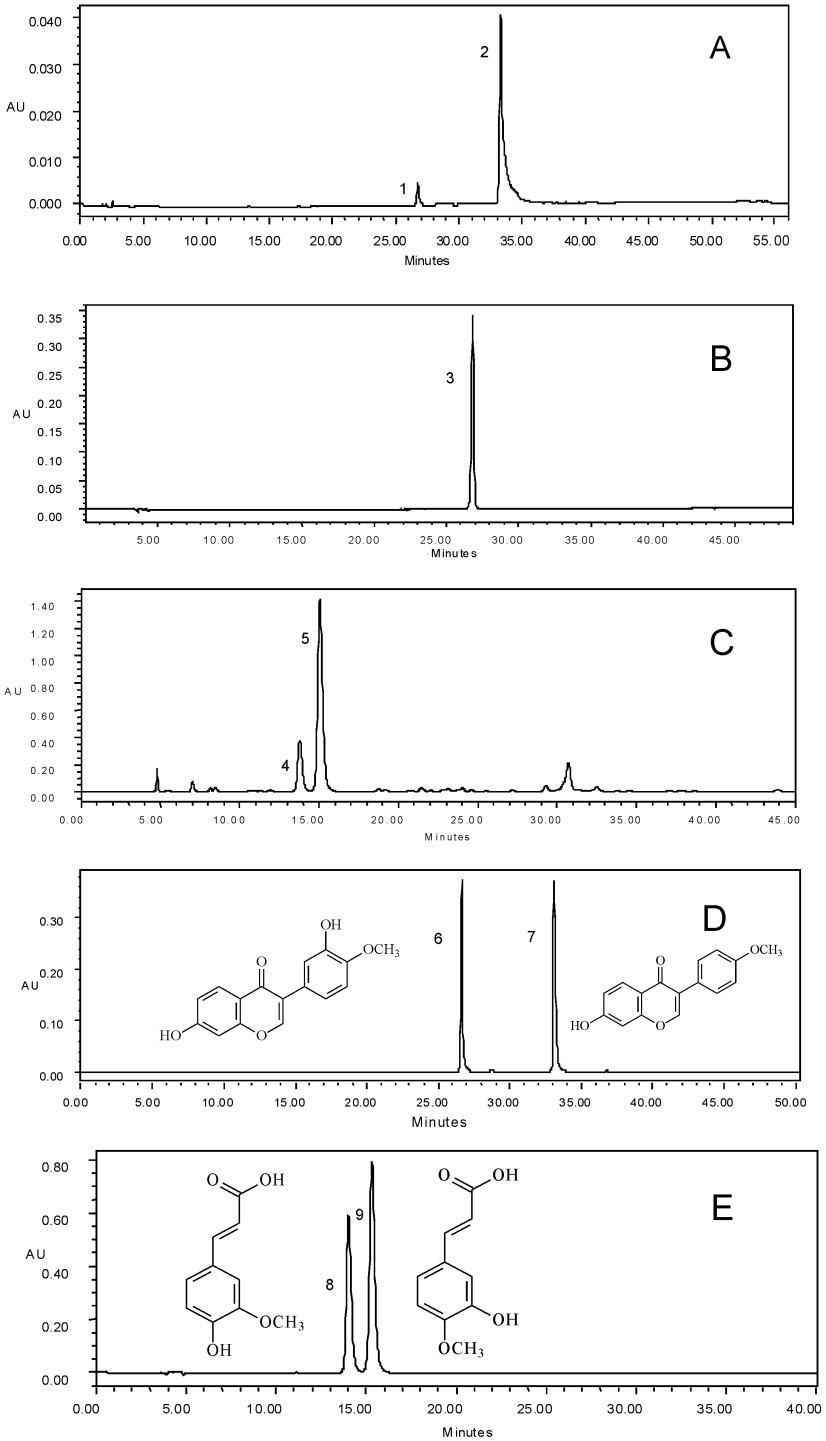
HPLC spectrogram and structures of synergistic antioxidants. A: Spectrogram of purified sample from AME-CF_2_, B: Spectrogram of purified sample from AME-CF_4_, C: Spectrogram of purified sample from CFO-CF_1_. D: Spectrogram of mix standard solution of calycosin and formononetin, peak 6 was calycosin, peak 7 was formononetin. E: Spectrogram of mix standard solution of ferulic acid and isoferulic acid, peak 8 was ferulic acid, peak 9 was isoferulic acid.

These components were further characterized by HPLC-ESI-MS/MS, and their MS/MS spectra, retention time and UV λ_max_ were shown in Table 4. Based on the comparison with standard solutions, the antioxidant components were unambiguously identified as calycosin (1), formononetin (2) in AME-CF_2_, calycosin (3) in AME-CF_4_, and ferulic acid (4), isoferulic acid (5) in CFO-CF_1_. Their structures were listed in Fig. 4.

**Table 4:** Data from HPLC-DAD-ESI-MS/MS for characterization of potential synergistic antioxidants.

Peak^a^	*t*_R_ a (min)	[M+H]^+^ (m/z)	[M+Na]^+^ (m/z)	MS/MS (m/z)	UV λ_max_(nm)	Assigned identity
1, 3	26.712/26.340	285	307	307,270,253, 225	249,291	Calycosin
2	33.259	269	291	291,253,226, 198	248,301	Formononetin
4	13.782	195	–b	177,149,117, 134,145	217,235,324	Ferulic acid
5	15.040	195	–	177,149,117, 134	217,241,322	Isoferulic acid

a numbers and retention time refer to Figure 4.

b not detected.

### Verification of the Synergistic Combination using DPPH Radical Scavenging Assay

**Figure 5:**
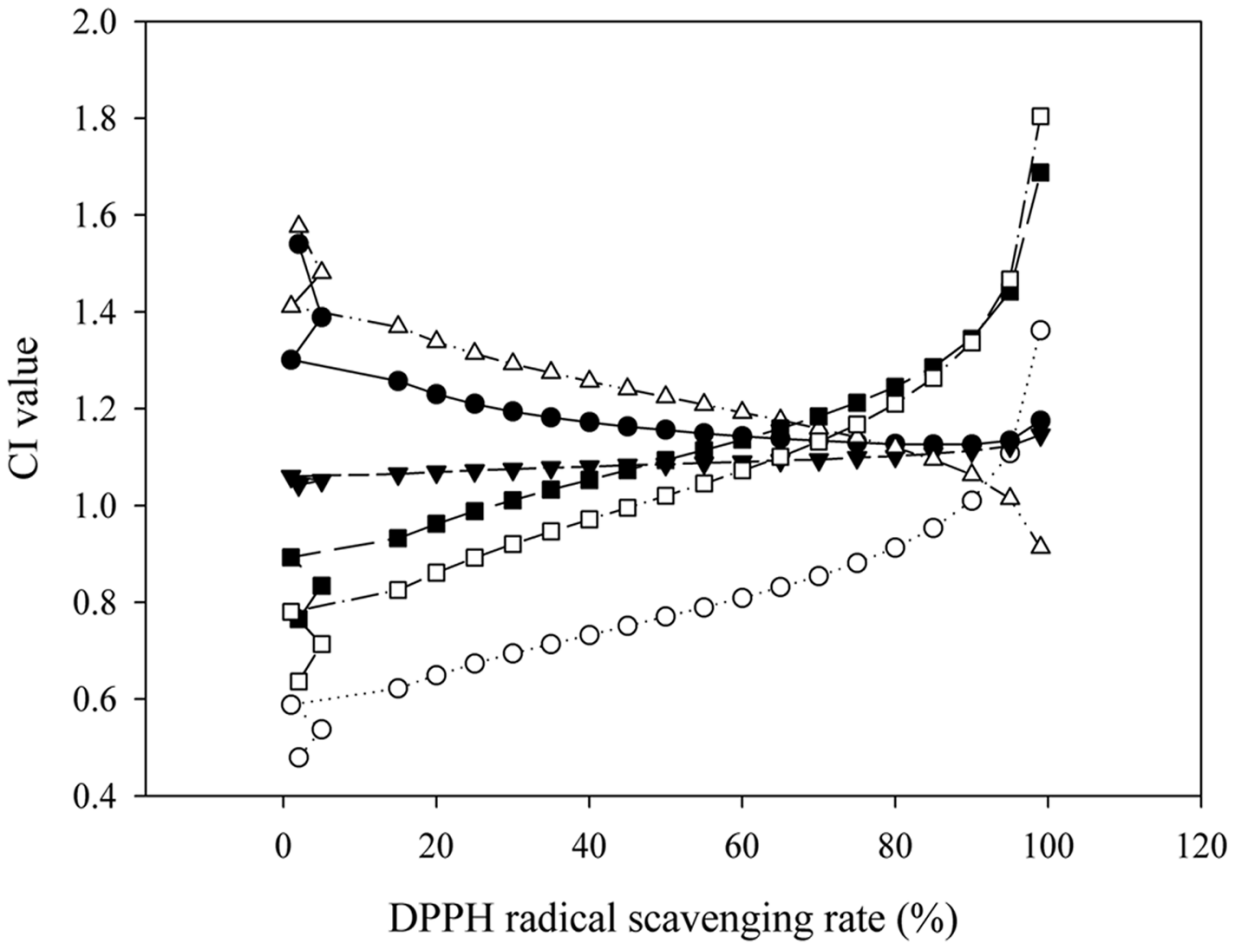
The CI simulations for different combinations of standard substances. • Ferulic acid/calycosin, 1∶1; ○ Isoferulic acid/calycosin, 1∶1; ▾ Isoferulic acid/calycosin, 1∶2; △ Isoferulic acid/calycosin, 1∶3; ▪ Isoferulic acid/calycosin, 3∶1; □ Isoferulic acid/calycosin, 2∶1.

In DPPH radical scavenging assay, the poor scavenging activity was observed in formononetin with a percent inhibition of 0.72% at the concentration of 8.05 mg/mL. The IC_50_ of calycosin was 0.64 mg/mL. These results might suggest that the potent free radical scavenging abilities of AME-CF_2_ and AME-CF_4_ were greatly attributed to calycosin. Therefore, calycosin was selected to combine with ferulic acid (IC_50_, 0.26 mg/mL) and isoferulic acid (IC_50_, 1.50 mg/mL) respectively to examine their synergistic effects. The results from Fig. 5 showed that the combination of calycosin and ferulic acid exhibited slight antagonism (CI_50_, 1.16), while the combination of calycosin and isoferulic acid showed synergistic effect (CI_50_, 0.77). In order to observe whether the dose ratio could affect the synergistic effect between calycosin and isoferulic acid, different dose ratios were tested and the strongest synergy was observed at a dose ratio of 1∶1. Furthermore, with the reduction of the dosage, the radical scavenging ability decreased, while the synergistic effect of this combination was improved.

### ABTS Radical Scavenging Ability and FRAP Value of the Potential Synergistic Compounds

ABTS radical scavenging ability and ferric reducing antioxidant capacity of these four potential synergistic antioxidant compounds were also measured in order to evaluate their synergistic effects from different aspects. The results in Table 5 indicated that these compounds exhibited strong radical scavenging ability and ferric reducing antioxidant capacity with an exception of formononetin. In FRAP assay, the synergistic effect was observed in the combination of isoferulic acid and calycosin. While, as for ABTS radical scavenging assay, the combination of isoferulic acid and calycosin only showed additive effect (CI_50_, 1.062), and the other combinations even exhibited slight antagonism.

**Table 5:** ABTS radical scavenging ability, ferric reducing antioxidant activity and synergistic effect of different samples.

Samples	IC_50_(ABTS) (mg/mL)	CI_50_ (mg/mL)	FRAP value (mmol/g)	Theoretical value (mmol/g)
FA^a^	0.061		8.400	
IFA^b^	0.109		11.424	
FOR^c^	7.468		–	
CAL^d^	0.159		4.905	
FA+ FOR	0.133	1.263		
FA+ CAL	0.111	1.230		
IFA+ FOR	0.278	1.148	6.699	6.652
IFA+ CAL	0.117	1.062	8.706*	8.164

a Ferulic acid,

b Isoferulic acid,

c Formononetin,

d Calycosin.

* There was a significant difference between the actual FRAP value and the theoretical value (*P*<0.05).

### Protective Effect of Potential Synergistic Compounds against H_2_O_2_-induced Oxidative Damage in HepG2 Cells

**Figure 6:**
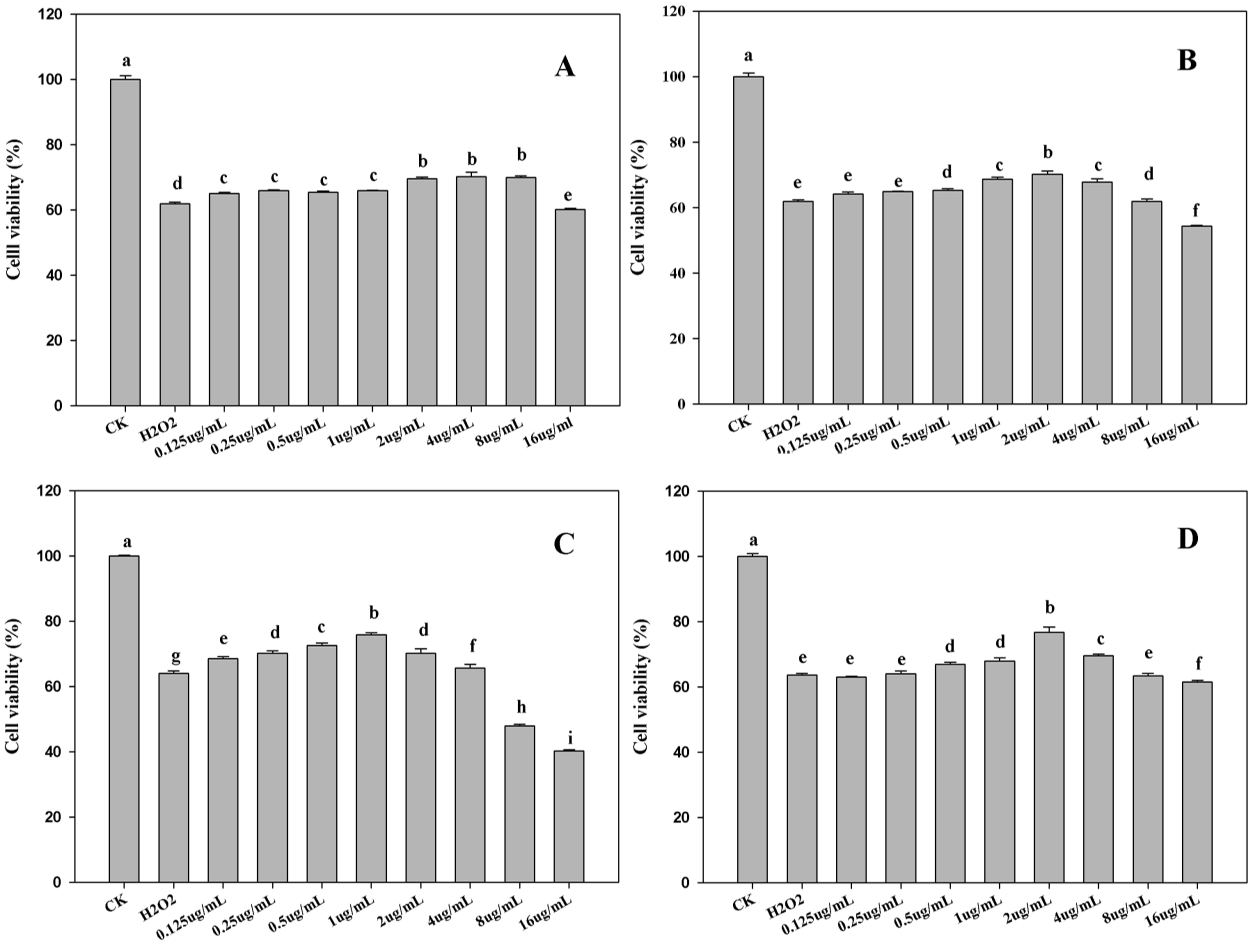
The cell viability of potential synergistic antioxidant compounds. A: The cell viability of ferulic acid in different concentrations. B: The cell viability of isoferulic acid in different concentrations. C: The cell viability of formononetin in different concentrations. D: The cell viability of calycosin in different concentrations.

Many natural antioxidants that provide an antioxidant effect i*n vitro* may have a pro-oxidant effect *in vivo* or in cultured cells, so it is necessary to proceed to *in vivo* assays after evaluating antioxidant activities *in vitro* using chemical-based methods. The animal models and clinical studies are advanced but expensive and time-consuming, making the cell-based assays exceedingly attractive [22]. Three concentration gradients were screened from each drug according to the results in Fig. 6, i.e. 2 µg/mL, 1 µg/mL, 0.5 µg/mL for Ferulic acid, isoferulic acid and calycosin, and 1 µg/mL, 0.5 µg/mL, 0.25 µg/mL for formononetin. Therefore, nine combinations of herbal pair were examined for their synergistic effects on protecting cells against H_2_O_2_-induced oxidative damage. As shown in Table 6 and Table 7, the results indicated that the combination of isoferulic and calycosin exhibited synergistic effect at a dose of 1∶1 (CI, 0.442) and 2∶1 (CI, 0.636) in HepG2 cell-based assay. Furthermore, the synergy was also observed in the other combinations (Ferulic acid+Formononetin, Ferulic acid+Calycosin, Isoferulic acid+Formononetin) when they were combined at appropriate dose ratio.

**Table 6:** Cell protective ability and the synergistic effect for the combination of Ferulic acid and Formononetin, Calycosin.

Samples (µg/mL)	Fractionaffected (Fa)^d^	CI value
FA^a^ (1)	0.131	
FOR^b^ (0.25)	0.103	
FA+FOR (FA: 0.5, FOR: 0.125)	0.424	0.424
FA (0.5)	0.103	
FA (2)	0.228	
CAL^c^ (2)	0.343	
CAL (1)	0.152	
FA+CAL (FA: 0.25, CAL: 1)	0.335	0.526
FA+CAL (FA: 0.25, CAL: 0.5)	0.182	0.656
FA+CAL (FA: 1, CAL: 0.5)	0.220	0.877

a Ferulic acid,

b Formononetin,

c Calycosin.

d
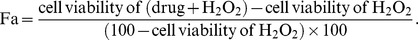

**Table 7:** Cell protective ability and the synergistic effect for the combination of Isoferulic acid and Formononetin, Calycosin.

Samples (µg/mL)	Fractionaffected (Fa)^d^	CI value
IFA^a^ (0.5)	0.103	
FOR^b^ (0.5)	0.143	
FOR (0.25)	0.100	
IFA+FOR (IFA: 0.25, FOR: 0.25)	0.252	0.314
IFA+FOR (IFA: 0.25, FOR: 0.125)	0.273	0.193
IFA^c^ (0.5)	0.070	
IFA (1)	0.172	
CAL (0.5)	0.078	
IFA+CAL (IFA: 0.25, CAL: 0.25)	0.168	0.442
IFA+CAL (IFA: 0.5, CAL: 0.25)	0.172	0.636

a Isoferulic acid,

b Formononetin,

c Calycosin.

a Isoferulic acid,

b Formononetin,

c Calycosin.

d
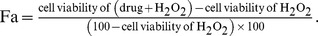

## Discussion

Free radicals are highly unstable and tend to induce oxidative damage to other molecules by extracting electron, then cause various diseases such as inflammation, atherosclerosis, and aging [23]. Therefore, antioxidants with free radical scavenging activities may play a significant role in the prevention and therapeutics of these diseases. As we all know, phenolic acid and flavonoid compounds are important radical scavengers. In this study, the correlation analysis between the total phenolic/flavonoid increments and the enhancement of antioxidant activities demonstrated the significant contribution of the phenolic/flavonoid compounds to the antioxidant efficacy of AME and CFO. Extraction with different polar solvents is a common method used for the separation of botanical compositions. The bioactive compounds such as phenolic acids and flavonoids were generally enriched in chloroform extracts and ethyl acetate extracts. Yu et al. [24]isolated and identified 10 compounds from different solvent-extracted fractions of Huang-qi, and found that calycosin in ethyl acetate extracted fraction exhibited stronger DPPH radical scavenging ability than ononin from n-butyl alcohol extracted fraction. Calycosin also existed in the chloroform extracted fraction of Huang-qi [25]. Li et al. [26] proved the significant contribution of phenolics to the antioxidant ability of Sheng-ma, and indicated that phenolic compounds were mainly obtained from ethyl acetate extracted fraction, while saponins were generally present in the petroleum ether extracted fraction.

Up to date, a variety of phenolic compounds have been extensively investigated for their antioxidant activity. However, to our knowledge, these studies mainly focused on the single phenolic compound, the informations regarding the interactive actions among them are still limited. Peng et al. isolated ten flavonoid compounds from *Polygonum hydropiper* L., and found that there was not synergistic anti-oxidative capacity when the flavonoid compounds were used in combination [27]. Other recent study on phenolic compounds (chlorogenic acid, hesperidin, luteolin, myricetin, naringenin, *p*-coumaric acid, and quercetin) of navel oranges showed that three combinations of 2 compounds (e.g. hesperidin/myricetin) and five combinations of 3 compounds (e.g. hesperidin/chlorogenicacid/naringenin) represented synergistic effects [4]. In our study, the combination of calycosin and isoferulic acid at a dose ratio of 1∶1 resulted in a significant synergy in DPPH radical scavenging assay, FRAP assay and HepG2 cells bioassay. Both isoferulic acid and calycosin possess hydroxyl groups, after being combined, the number of free hydroxyl groups may be increased and promote access of the free radical scavengers to the radical center [28]. Therefore, the concentration of the potential antioxidant components may be elevated and then appeared synergistic effect. Although isoferulic acid owned poorer DPPH free radical scavenging ability than ferulic acid, it showed antioxidant synergism when used in the combination with calycosin. This fact demonstrated that the combination of two strong antioxidants may not necessarily engender stronger efficacy, it may even produce antagonistic interaction. In contrast, when a weaker antioxidant was combined with a stronger one, the former may regenerate the latter, thus improving the overall free radical quenching capacity of the combination [29]. Dai et al. revealed that polyphenols in green tea could regenerate V_E_, and L-ascorbic acid could accelerate the regenerate of oxidized polyphenols in green tea, then formed an antioxidant cycle system [30]. Furthermore, ingredients which possess different antioxidant mechanisms also could exhibit synergistic effect. For example, quercetin as a metal chelating agent could support a-tocopherol to improve the antioxidant ability of oil [31]. Likewise, isoferulic acid has been found to be a metal ion chelating agent [32], which might help calycosin to strengthen the antioxidant activities of the combination. The combination of isoferulic acid and calycosin at low dose showed better synergistic effect than at a high dose. This fact indicated that the synergistic interactions may be weakened and even can be transformed into antagonistic interactions under a higher dose, then to bring about adverse effects on body.

In addition, although these obtained compounds showed potent synergistic effect, this effect was weakened to some extent when compared with the original samples prior to purification. These findings may indicate that some components which contributed to the synergistic effect might be separated into other fractions. It also demonstrated the complexity of synergistic antioxidants when the edible and medicinal plants were utilized in the combination. The fact that the synergistic combination screened based on DPPH radical scavenging assay did not exhibit similar synergy in scavenging ABTS radicals demonstrated the difference between various antioxidant methods. Not a single method can give a comprehensive prediction of antioxidant efficacy, so more than one *in vitro* antioxidant method should be applied to screening the synergistic compounds from plants. Since most of the chemical assays are done in non-physiological pH values, the results of chemical assays and *in vivo* models may be inconsistent. In this paper, more than one synergistic combination was observed in cell-based assays, suggesting that the *in vitro* chemical methods are convenient but clearly limited. Thus, in order to better elucidate the mechanism behind the antioxidant synergism of natural product, further cells bioassay and *in vivo* research should be done to study the bioavailability of the potential antioxidant like metabolism, uptake and portioning in membranes.

## References

[1] Sun J, Liu S-f, Zhang C-s, Yu L-n, Bi J, et al (2012) Chemical composition and antioxidant activities of Broussonetia papyrifera fruits. PloS one 7: e32021. 22389678 10.1371/journal.pone.0032021 PMC3289642

[2] Li H-B, Wong C-C, Cheng K-W, Chen F (2008) Antioxidant properties in vitro and total phenolic contents in methanol extracts from medicinal plants. LWT-Food Science and Technology 41: 385–390.

[3] Williamson E (2001) Synergy and other interactions in phytomedicines. Phytomedicine 8: 401–409. 11695885 10.1078/0944-7113-00060

[4] Freeman BL, Eggett DL, Parker TL (2010) Synergistic and antagonistic interactions of phenolic compounds found in navel oranges. Journal of food science 75: C570–C576. 20722912 10.1111/j.1750-3841.2010.01717.x

[5] Prior RL, Wu X (2013) Diet Antioxidant Capacity: Relationships to Oxidative Stress and Health. Am J Biomed Sci 5: 126–139.

[6] Facino RM, Carini M, Aldini G, Calloni M, Bombardelli E, et al (2007) Sparing effect of procyanidins from Vitis vinifera on vitamin E: in vitro studies. Planta medica 64: 343–347. 10.1055/s-2006-957448 9619118

[7] Goupy P, Vulcain E, Caris-Veyrat C, Dangles O (2007) Dietary antioxidants as inhibitors of the heme-induced peroxidation of linoleic acid: Mechanism of action and synergism. Free Radical Biology and Medicine 43: 933–946. 17697938 10.1016/j.freeradbiomed.2007.06.013

[8] Liu D, Shi J, Colina Ibarra A, Kakuda Y, Jun Xue S (2008) The scavenging capacity and synergistic effects of lycopene, vitamin E, vitamin C, and *β*-carotene mixtures on the DPPH free radical. LWT-Food Science and Technology 41: 1344–1349.

[9] Palafox-Carlos H, Gil-Chávez J, Sotelo-Mundo RR, Namiesnik J, Gorinstein S, et al (2012) Antioxidant Interactions between Major Phenolic Compounds Found in ‘Ataulfo’Mango Pulp: Chlorogenic, Gallic, Protocatechuic and Vanillic Acids. Molecules 17: 12657–12664. 23103532 10.3390/molecules171112657 PMC6268240

[10] Guo F-Q, Li A, Huang L-F, Liang Y-Z, Chen B-M (2006) Identification and determination of nucleosides in Cordyceps sinensis and its substitutes by high performance liquid chromatography with mass spectrometric detection. Journal of Pharmaceutical and Biomedical Analysis 40: 623–630. 16168606 10.1016/j.jpba.2005.07.034

[11] Yang WJ, Li DP, Li JK, Li MH, Chen YL, et al (2009) Synergistic antioxidant activities of eight traditional Chinese herb pairs. Biological and Pharmaceutical Bulletin 32: 1021–1026. 19483308 10.1248/bpb.32.1021

[12] Fu S, Zhang J, Menniti-Ippolito F, Gao X, Galeotti F, et al (2011) Huangqi injection (a traditional Chinese patent medicine) for chronic heart failure: a systematic review. PloS one 6: e19604. 21573109 10.1371/journal.pone.0019604 PMC3089614

[13] Pan R-L, Chen D-H, Si J-Y, Zhao X-H, Li Z, et al (2009) Immunosuppressive effects of new cyclolanostane triterpene diglycosides from the aerial part of Cimicifuga foetida. Archives of pharmacal research 32: 185–190. 19280146 10.1007/s12272-009-1133-1

[14] Brand-Williams W, Cuvelier M, Berset C (1995) Use of a free radical method to evaluate antioxidant activity. LWT-Food Science and Technology 28: 25–30.

[15] Rathee JS, Patro BS, Mula S, Gamre S, Chattopadhyay S (2006) Antioxidant activity of Piper betel leaf extract and its constituents. J Agric Food Chem 54: 9046–9054. 17117789 10.1021/jf061679e

[16] Wang C, Chang S, Inbaraj BS, Chen B (2010) Isolation of carotenoids, flavonoids and polysaccharides from *Lycium barbarum* L. and evaluation of antioxidant activity. Food Chemistry 120: 184–192.

[17] Benzie IFF, Strain J (1996) The ferric reducing ability of plasma (FRAP) as a measure of “antioxidant power”: the FRAP assay. Analytical biochemistry 239: 70–76. 8660627 10.1006/abio.1996.0292

[18] Singleton V, Rossi JA (1965) Colorimetry of total phenolics with phosphomolybdic-phosphotungstic acid reagents. American journal of Enology and Viticulture 16: 144–158.

[19] Zhishen J, Mengcheng T, Jianming W (1999) The determination of flavonoid contents in mulberry and their scavenging effects on superoxide radicals. Food Chemistry 64: 555–559.

[20] Li M, Xu Y, Yang W, Li J, Xu X, et al.. (2011) In vitro synergistic anti-oxidant activities of solvent-extracted fractions from Astragalus membranaceus and Glycyrrhiza uralensis. LWT-Food Science and Technology.

[21] Kumaran A, Joel Karunakaran R (2007) In vitro antioxidant activities of methanol extracts of five *Phyllanthus* species from India. LWT-Food Science and Technology 40: 344–352.

[22] López-Alarcón C, Denicola A (2012) Evaluating the antioxidant capacity of natural products: A review on chemical and cellular-based assays. Analytica chimica acta. 10.1016/j.aca.2012.11.051 23340280

[23] Costa RM, Magalhães AS, Pereira JA, Andrade PB, Valentão P, et al (2009) Evaluation of free radical-scavenging and antihemolytic activities of quince (*Cydonia oblonga*) leaf: A comparative study with green tea (*Camellia sinensis*). Food and Chemical toxicology 47: 860–865. 19271320 10.1016/j.fct.2009.01.019

[24] Yu D, Bao Y, Wei C, An L (2005) Studies of chemical constituents and their antioxidant activities from Astragalus mongholicus Bunge. Biomedical and Environmental Sciences 18: 297. 16370311

[25] Lin L-Z, He X-G, Lindenmaier M, Nolan G, Yang J, et al (2000) Liquid chromatography–electrospray ionization mass spectrometry study of the flavonoids of the roots of *Astragalus mongholicus* and *A. membranaceus* . Journal of Chromatography A 876: 87–95. 10823504 10.1016/s0021-9673(00)00149-7

[26] Li X, Lin J, Gao Y, Han W, Chen D (2012) Antioxidant activity and mechanism of Rhizoma Cimicifugae. Chemistry Central Journal 6: 1–10. 23173949 10.1186/1752-153X-6-140 PMC3557226

[27] Peng ZF, Strack D, Baumert A, Subramaniam R, Goh NK, et al (2003) Antioxidant flavonoids from leaves of *Polygonum hydropiper* L. Phytochemistry. 62: 219–228. 10.1016/s0031-9422(02)00504-6 12482460

[28] Cai Y-Z, Sun M, Xing J, Luo Q, Corke H (2006) Structure–radical scavenging activity relationships of phenolic compounds from traditional Chinese medicinal plants. Life Sciences 78: 2872–2888. 16325868 10.1016/j.lfs.2005.11.004

[29] Peyrat-Maillard M, Cuvelier M, Berset C (2003) Antioxidant activity of phenolic compounds in 2, 2′-azobis (2-amidinopropane) dihydrochloride (AAPH)-induced oxidation: Synergistic and antagonistic effects. Journal of the American Oil Chemists’ Society 80: 1007–1012.

[30] Dai F, Chen W-F, Zhou B (2008) Antioxidant synergism of green tea polyphenols with α-tocopherol and l-ascorbic acid in SDS micelles. Biochimie 90: 1499–1505. 18554517 10.1016/j.biochi.2008.05.007

[31] Hudson BJ, Lewis JI (1983) Polyhydroxy flavonoid antioxidants for edible oils. Phospholipids as synergists. Food Chemistry 10: 111–120.

[32] Wang X, Li X, Chen D (2011) Evaluation of antioxidant activity of isoferulic acid in vitro. Natural product communications 6: 1285. 21941899

